# Fungal Nail Infections (Onychomycosis): A Never-Ending Story?

**DOI:** 10.1371/journal.ppat.1004105

**Published:** 2014-06-05

**Authors:** Mahmoud Ghannoum, Nancy Isham

**Affiliations:** Center for Medical Mycology, University Hospitals of Cleveland, Cleveland, Ohio, United States of America; The University of North Carolina at Chapel Hill, United States of America

## Is Onychomycosis Still a Problem?

**Figure 1:**
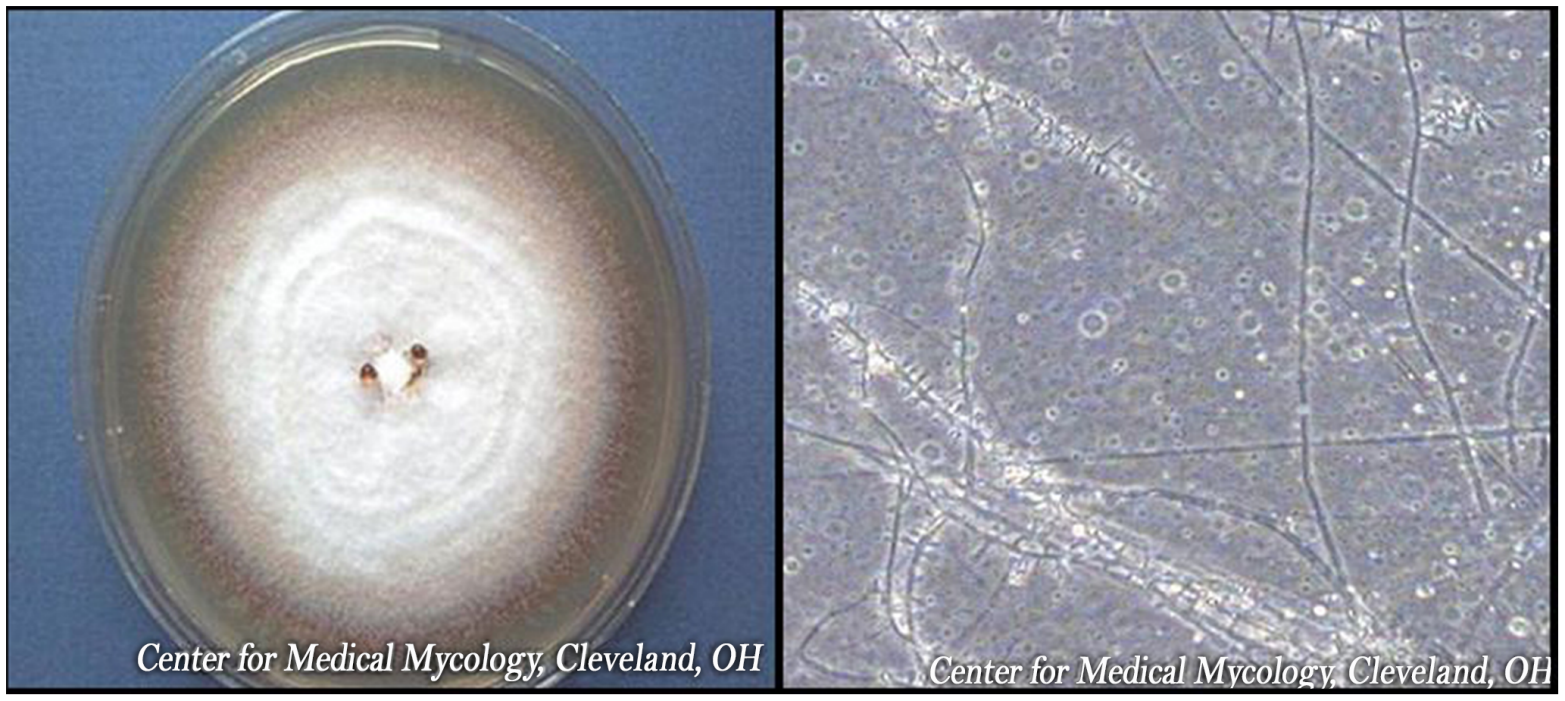
*Trichophyton rubrum* colony and microscopic appearance (40x).

**Figure 2:**
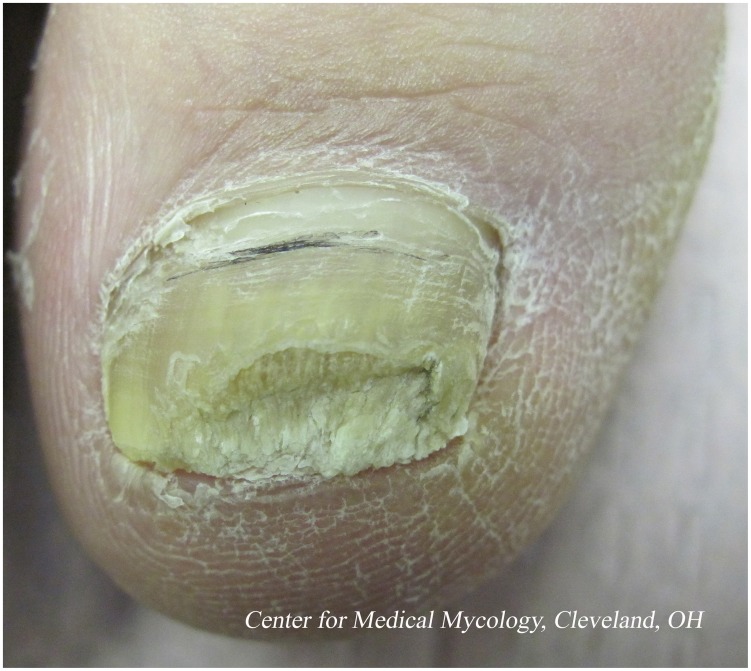
Distal subungual onychomycosis of the great toenail.

The great majority of superficial fungal infections are caused by dermatophytes, which belong to one of three genera (*Trichophyton, Epidermophyton*, and *Microsporum*), with *T. rubrum* being the most prominent cause of nail infection (Figure 1). Table 1 summarizes the prevalence of various superficial fungal infections in different geographic areas [1]. Among superficial fungal infections, by far the most difficult to cure is toenail onychomycosis (Figure 2). The prevalence of onychomycosis has been reported to be as high as 23% across Europe [2] and 20% in East Asia [3]. In North America, the incidence of onychomycosis is up to 14% [4], with fungal infection responsible for 50% of all nail disease [5]. With millions of dollars being spent annually on oral and topical prescriptions, laser treatments, over-the-counter products, and home remedies, it is obvious that people are still bothered by their fungal toenail infections and are determined to get rid of them. Unfortunately, this is easier said than done. To successfully cure toenail onychomycosis requires long treatment duration that may extend to a full year. Even then, complete cure, defined as clinical cure (implying nail clearing) plus mycological cure (both negative microscopy and dermatophyte culture), is often unattainable.

**Table 1:** Most prevalent dermatophytosis in different regions worldwide.

Region	Dermatophytosis	Causative Organism
North/South America	Tinea pedis, onychomycosis	*T. rubrum*
Western Europe	Tinea pedis, onychomycosis	*T. rubrum*
Russia	Onychomycosis	*T. rubrum*
Mediterranean (Italy/Greece)	Tinea corporis, tinea capitis	*M. canis*
Turkey	Tinea pedis	*T. rubrum*
North Africa/tropical Africa	Tinea corporis	*T. violaceum, M. audouinii*
China/Japan	Tinea pedis, onychomycosis	*T. rubrum*
India	Tinea corporis	*T. rubrum*
Asia	Tinea pedis, onychomycosis	*T. rubrum, T. mentagrophytes*
Australia	Tinea pedis, onychomycosis	*T. rubrum, T. mentagrophytes*

*Data for this table was compiled from Havlickova et al. [1].

## What Are the Risk Factors for Toenail Onychomycosis?

The most prevalent predisposing risk factor for developing onychomycosis is advanced age, which is reported to be 18.2% in patients 60–79 years of age, compared to 0.7% in patients younger than 19 years of age. Further, men are up to three times more likely to have onychomycosis than women, though the reasons for this gender difference are not clear [6]. Moreover, the low prevalence of infection in people whose spouses have onychomycosis compared to the prevalence among their children suggests a genetic risk factor [7]. Though extremely rare, one study reported four family members from seven unrelated groups with a common genetic trait (autosomal recessive CARD9 deficiency) who developed a dermatophyte infection of deep tissues that proved fatal [8].

Other risk factors include diabetes and conditions contributing to poor peripheral circulation [9]. In fact, onychomycosis may represent an important predictor for the development of diabetic foot syndrome and foot ulcers [10]. Patients who are immunosuppressed, such as those with HIV infection and those undergoing cancer therapy, are also predisposed to fungal nail infection [11]. There is at least one case report of a toenail infection caused by *Fusarium* (a non-dermatophyte fungus) that developed into a fatal systemic infection in a lymphoma patient following a bone marrow transplant [12].

Several nonclinical risk factors also affect a person's chance of developing fungal nail infections. Toenail onychomycosis is not prevalent in tropical climates, presumably because people in those areas are not in the habit of wearing occlusive footwear that create a warm, moist environment for the proliferation of fungi. Further, the spread of foot infections, including tinea pedis (athlete's foot), may occur in places such as shower stalls, bathrooms, or locker rooms where floor surfaces often are wet and people are barefoot [13]. Nail trauma will also increase the risk of fungal infection of the affected nail, especially in the geriatric population [11].

A recent study by our group utilized regression analysis to show that history of tinea pedis plus three clinical variables—onychomycosis, plantar scaling (a clinical sign of tinea pedis), and nail discoloration (a clinical sign of onychomycosis and generally indicative of severe nail infection) were statistically associated with spread of infection in households with multiple infected members (*P*≤.044) [14].

## How Is Onychomycosis Treated?

Treatment of onychomycosis includes chemical or surgical removal of the infected nail, systemic or topical drugs, pulse therapy, or a combination thereof. Table 2 is a summary of oral and topical therapy regimens; as can be seen, the course of treatment for fingernail infections is shorter than for toenail infections. The treatment of onychomycosis has improved considerably over the past several decades, following the introduction of the oral antifungals terbinafine and itraconazole. However, these drugs may have side effects such as liver damage or drug interactions, which are particularly relevant in the elderly population [15]. Further, nail infections caused by non-dermatophyte organisms, such as *Fusarium*, are especially difficult to treat [16].

**Table 2:** Treatment of onychomycosis with antifungal agents.

Agent	Dose	Duration
Terbinafine	250 mg	Toenails: once per day for 12 weeks
		Fingernails: once per day for 6 weeks
Itraconazole	200 mg	Toenails: once per day for 12 weeks
	pulse therapy	Toenails: 200 mg twice per day for 1 week/no treatment for 3 weeks. Repeat for 3–4 months
		Fingernails: 200 mg twice per day for 1 week/no treatment for 3 weeks. Repeat for 2 months
Fluconazole	300–450 mg	Toenails: once/week for 9–12 months
	150–300 mg	Fingernails: once/week for 4–6 months
Ciclopirox nail lacquer	apply once per day	Remove lacquer once per week. Treat for up to 48 weeks
Amorolfine nail lacquer	apply once or twice a week	Remove lacquer before each new application. Toenails: 9–12 months. Fingernails: 6 months

## Why Don't Topical Antifungals Work Better?

Unfortunately, currently available topical agents, such as amorolfine 5% and ciclopirox 8%, have low efficacy (approximately 5%–12%) [17], [18]. This low efficacy can mainly be attributed to the inability of the drug to penetrate through the nail plate to the nail bed where the infection resides [19]. Thickened nails, extensive involvement of the entire nail, lateral disease, and yellow spikes contribute to a poor response to topical treatment [11]. Figure 1 shows an example of distal subungual onychomycosis, trimmed to demonstrate nail thickening.

Further complicating the scenario is the fact that certain antifungals will bind to the nail plate and thus may not be available at the site of infection, which is the nail bed. For example, terbinafine has been shown to accumulate rapidly in the nail, reaching a maximum of 0.39 mg/g and persisting up to 2 months following the end of treatment [20]. In this regard, Ryder et al. developed an in vitro nail model that showed that the cidal action of terbinafine, when tested against an established dermatophyte infection in the presence of human nail, was in fact less effective than in conventional microdilution assays where no nail powder is present [21].

Many different approaches to solving the problem of nail penetration have been attempted recently. For example, there have been attempts to develop penetration enhancers to facilitate drug delivery through the nail plate, such as addition of dodecyl-2-N,N-dimethylaminopropionate hydrochloride (DDAIP HCl, trade name NexACT-88; NexMed) to terbinafine nail lacquer [19]. Another technique to enhance the penetration of nail lacquer was the incorporation of terbinafine into transfersome lipid vesicles, which are highly deformable and thus are able to pass through intercellular spaces [22]. Additionally, a novel small-molecule oxaborole antifungal (AN2690) has recently been developed that is designed for greater penetration through the nail plate [23]. However, to date, none of these topical products has been commercialized.

In this regard, approval of topical onychomycosis drugs by regulatory agencies may be negatively impacted by an overly stringent definition of complete cure, which includes nail clearing plus mycological cure (negative microscopy and culture). Review of data from several international clinical trials by Ghannoum et al. has suggested that reassessment of the definition of onychomycosis cure is critical [24]. In these trials, a high number of toenail samples collected from subjects at the end of clinical trials contained visible fungal hyphae that subsequently failed to grow upon culture. However, current technology does not differentiate between “live” and “dead” fungi, so even though these samples had to be reported as microscopy-positive, the infection may in fact have been cured. The authors propose that, for clinical trials of topical agents, the length of treatment should be extended to 18 months, followed by a longer washout period of 3–6 months before primary assessments to allow the removal of both residual drug in the nail and nonviable fungal cells. Therefore, the absence of clinical signs following an adequate wash out period, coupled with a negative culture, with or without negative microscopy, should be considered the definition of onychomycosis cure. These changes may enable more topical agents to be proven efficacious.

## What's New in Onychomycosis Therapy?

Recent device-based therapies for onychomycosis include laser devices, photodynamic therapy, iontophoresis, and ultrasound. Laser treatment has been approved for cosmetic treatment only, but efficacy as a treatment to eradicate the fungal infection will have to be determined by additional randomized controlled trials [25]. There have been rare reports of onychomycosis cures following the use of phototherapy, which involves the irradiation of accumulated protoporphyrin within the fungal hyphae, leading to subsequent hyphal cell damage [26]. The ability of iontophoresis, or the use of electric current (0.5 mA/cm^2^) to facilitate the passage of drug through the nail plate, has been proven in studies with human cadaver nails, but relevant clinical studies remain to be conducted [27]. Finally, though ultrasound therapy has preliminarily demonstrated fungistatic activity against nail infections [28], the device itself seems overly complicated, with ultrasound transducers and drug delivery compartments needed above each toenail and the requirement for a computer software interface, making it a physician-office–only treatment and likely very expensive [29].

## Why Do Patients So Often Relapse?

There are multiple factors that may contribute to the high rate of fungal nail infection recurrence. Patients with a genetic predisposition to onychomycosis, who are immunocompromised, or who have diabetes, are likely to experience relapse and may never achieve a permanent cure [11].

This may be due either to failure to eradicate the infecting fungus or to re-infection with a new fungal strain following subsequent exposure. Arthroconidia, which are chains of fungal conidia that are formed by breakage of the fungal hyphae, are considered to be the primary means of nail invasion. These arthroconidia, which have thicker cell walls than conidia formed in vitro, have been shown to be more resistant to antifungals and, thus, may remain in the nail bed as a reservoir for recurrent disease [30]. However, the incidence of innate resistance among dermatophytes is low. Our Center conducted in vitro susceptibility testing of 140 sequential isolates from subjects who failed treatment in an oral terbinafine clinical trial. In all cases, the minimum inhibitory concentrations (MICs) of terbinafine against each patient set were identical or within one tube dilution, implying no resistance development. The same results were obtained within each set with fluconazole, itraconazole, and griseofulvin (with the exception of one isolate having a 3-fold increase in the MIC). This further indicates that there was no crossresistance between antifungal agents [31]. This study showed that failure to cure the infected nails may be due to host/family factors.

Even in cases where the infecting fungus has been entirely eradicated by antifungal therapy, patients remain at risk for re-infection. As mentioned above, people are exposed to dermatophyte reservoirs in many of their day-to-day activities, including trips to the gym and increased travel. Common sense measures, such as not walking barefoot through public showers or hotel rooms, would help prevent unnecessary exposure. That being said, one of the most common routes of infection is within households. It has long been suspected that nail infections were spread by close contact with family members. However, it wasn't until recently that our group was able to employ molecular techniques to prove that persons within the same household were infected by the same strain of *T. rubrum* [14]. For those attempting to avoid re-infection, measures such as spraying their shoes with a topical antifungal spray or treating them with a commercial ultraviolet device [32] after each wearing would be prudent. Thus, a patient not only needs to treat the infection but also break the cycle of re-infection.
